# Scientific authorship and collaboration network analysis on malaria research in Benin: papers indexed in the web of science (1996–2016)

**DOI:** 10.1186/s41256-018-0067-x

**Published:** 2018-04-06

**Authors:** Roseric Azondekon, Zachary James Harper, Fiacre Rodrigue Agossa, Charles Michael Welzig, Susan McRoy

**Affiliations:** 1 Centre de Recherche Entomologique de Cotonou, Cotonou, Benin; 2 University of Wisconsin Milwaukee, Milwaukee, WI 53211 USA; 3 Medical College of Wisconsin, Milwaukee, WI 53226 USA

**Keywords:** Network analysis, Scientific collaboration, Co-authorship, Malaria, Benin

## Abstract

**Background:**

To sustain the critical progress made, prioritization and a multidisciplinary approach to malaria research remain important to the national malaria control program in Benin. To document the structure of the malaria collaborative research in Benin, we analyze authorship of the scientific documents published on malaria from Benin.

**Methods:**

We collected bibliographic data from the Web Of Science on malaria research in Benin from January 1996 to December 2016. From the collected data, a mulitigraph co-authorship network with authors representing vertices was generated. An edge was drawn between two authors when they co-author a paper. We computed vertex degree, betweenness, closeness, and eigenvectors among others to identify prolific authors. We further assess the weak points and how information flow in the network. Finally, we perform a hierarchical clustering analysis, and Monte-Carlo simulations.

**Results:**

Overall, 427 publications were included in this study. The generated network contained 1792 authors and 116,388 parallel edges which converted in a weighted graph of 1792 vertices and 95,787 edges. Our results suggested that prolific authors with higher degrees tend to collaborate more. The hierarchical clustering revealed 23 clusters, seven of which form a giant component containing 94% of all the vertices in the network. This giant component has all the characteristics of a small-world network with a small shortest path distance between pairs of three, a diameter of 10 and a high clustering coefficient of 0.964. However, Monte-Carlo simulations suggested our observed network is an unusual type of small-world network. Sixteen vertices were identified as weak articulation points within the network.

**Conclusion:**

The malaria research collaboration network in Benin is a complex network that seems to display the characteristics of a small-world network. This research reveals the presence of closed research groups where collaborative research likely happens only between members. Interdisciplinary collaboration tends to occur at higher levels between prolific researchers. Continuously supporting, stabilizing the identified key brokers and most productive authors in the Malaria research collaborative network is an urgent need in Benin. It will foster the malaria research network and ensure the promotion of junior scientists in the field.

## Background

Malaria remains one of the three major public health concerns in Sub Saharan Africa where it affects millions of people and impact negatively on their socioeconomic life [1]. In the Millenium Declaration, Malaria has been given a special attention in terms of the successful achievement of the 6th development goal of the Millenium Challenge [2]. In Benin, initiatives such as the US President’s Malaria Initiative have supported governmental and non-governmental organizations to reduce the mortality and morbidity related to Malaria [3, 4]. With these financial supports at hand, such efforts in Benin have led to a sharp increase in public health interventions and many positive public health outcomes in terms of the reduction of mortality and morbidity related to Malaria [5]. Such increase in public health interventions translated in the successful implementation and sustainability of entomological surveillance of malaria for more than six years since 2008 [6]. Between the years 2000 and 2009, the increase in funding led to an annual decrease of 5.2% in the incidence of malaria and 5.3% in malaria-related deaths [7]. This encouraging success stories have even motivated other authors to enunciate the ambitious malaria eradication plan [8].

Despite the progress in malaria, very little is known on the dynamics of the malaria research collaboration network. This situation results in a lack of information on the main players and drivers of the progress made. As for the eradication of chickenpox [9], collaborative research will undoubtedly play an important role in the successful attainment of the malaria eradication plan in Subsaharan Africa in general and in Benin in particular. By collaborating with each other, researchers form continuous and sustainable collaboration through intensive network practices that go beyond the regional boundaries [10]. In addition, the fact that the extensive research conducted has not prevented malaria from outpacing the proposed solutions is a definitive clue to investigating the structure of the malaria research community. Research collaboration constitutes a stable basis for the provision of evidence based information in the formulation of fundamental principles and guidelines for the elaboration of public health strategies. Therefore, we propose in this study, to document, describe and analyze the different aspects of the malaria research collaboration in Benin.

Understanding the structure of this network is capital since it can help improve research prioritization [11], identify prolific researchers, better design, strategic planning and implementation of research programs [12], and promote cooperation and translational research initiatives [13]. We choose a social network analysis approach which will reveal undiscovered knowledge on effort of researchers in working together towards the reduction of the burden of Malaria in Benin.

Our study focuses on the Network analysis of the scientific collaborations through co-authorship network analysis. Its aim is to document the structure of the malaria collaborative research in Benin.

## Methods

### Data collection

The data collection was carried on papers indexed in Thompson’s Institute for Scientific Information Web Of Science (formerly known as the Web of Knowledge). The search was conducted using combinations of Malaria related MeSH terms including “malaria”, “Anopheles”, “Plasmodium” and “vector”. We restricted the search to the period from 1996 to 2016 and to “Benin” for country. We further screened the papers in order to only select those published by Beninese authors, or papers published on Malaria from Benin. All published documents under considerations included at least one Author from Benin. No restriction was placed upon the document types. We first started querying with each term independently, we then combined the other terms so the query return the maximum number of results. The Full citations information containing the authors’ names, their institutional affiliations, the year of publication, as well as the number of times the document was cited were recorded as a bibliographic corpus in text format. After a second screening only research that have met the above listed inclusion criteria and that were published between January 1, 1996 and December 31, 2016 were selected in this study.

#### Text mining and network generation

From the bibliographic text files, we built a corpus of the published documents using Tethne v0.8, a python software for parsing bibliographic data. Using NetworkX [14], another python package, we generated an undirected multigraph co-authorship networks containing parallel edges. Vertices were defined by several attributes including name, affiliation, city, country, number of publication and total number of times cited. Edges too, had attributes associated with them such as a unique identifier, the number of times a pair of authors was cited and the number of publications of a pair of authors. We normalized and disambiguate the information collected such as researchers’ names, research center denominations, and any other information that appeared ambiguous.

#### Author name disambiguation

One common challenge in collecting bibliometric data is the matching problem. Multiple names can refer to the same author. A well-known approach to solving this issue is termed as Author Name Disambiguation (AND). While many AND methods have been reported in the literature [15, 16], we performed a fuzzy matching machine learning technique of AND. We used Dedupe, a python library to disambiguate authors’ names and assign a unique identification number to each author. We manually annotated 10% of the names and then trained the algorithm to automatically disambiguate the remaining of the entries. Dedupe is interactive and adjusts further annotations as the disambiguation process evolves. Dedupe is based on the work of Bilenko [17] and has been developed by Gregg Forest and Derek Eder. For more information on Dedupe, we refer the reader to the author’s Github repository available at https://github.com/dedupeio/dedupe. We evaluated our AND fuzzy matching machine learning method by computing Precision and recall metrics.

### Descriptive data analysis

Using **igraph**, a network analysis package developed in R, we computed the following vertex centrality measures:

- Degree of the vertices in the network defined as the number of ties to a given author. After converting the multigraph network in a weighted graph where weights are the number of authorships between two authors, the strength of the vertices was also computed.
- Betweenness: it is the number of shortest paths between alters that go through a particular author. It relates to the perspective that importance relates to where a vertex is located with respect to the paths in the network graph. According to Freeman [18], it is defined as:

1 $$ {c}_B(v)=\frac{\sigma \left(s,t|v\right)}{\sum \limits_{s\ne t\ne v\in V}\sigma \left(s,t\right)} $$

where *σ*(*s*, *t*| *v*) is the total number of shortest paths between *s* and *t* that pass through *v*, and *σ*(*s*, *t*) is the total number of shortest paths between *s* and *t* regardless of whether or not they pass through *v*.

- Closeness: the number of steps required for a particular author to access every other author in the network. It captures the notion that a vertex is central if it is close to many other vertices. Considering a network *G* = (*V*, *E*) where *V* is the set of vertices and *E*, the set of edges, the closeness centrality *c*_*Cl*_(*v*) of a vertex *v* is defined as:

2 $$ {c}_{Cl}(v)=\frac{1}{\sum \limits_{u\in V} dist\left(v,u\right)} $$

where *dist*(*v*, *u*) is defined as the geodesic distance between the vertices *u*, *v* ∈ *V*.

- Eigenvectors: degree to which an author is connected to other well connected authors in the network. It seeks to capture the idea that the more central the neighbors of a vertex are, the more central that vertex itself is. According to Bonacich [19] and Katz [20], the Eigenvector centrality measure is defined as:

3 $$ {c}_{E_i}(v)=\alpha \sum \limits_{\left\{u,v\right\}\in E}{c}_{E_i}(u) $$

Where the vector ${\mathbf{c}}_{E_i}={\left({c}_{E_i}(1),\dots, {c}_{E_i}\left({N}_v\right)\right)}^T$ is the solution to the eigenvalue problem ${\mathbf{Ac}}_{E_i}={\alpha}^{-1}{\mathbf{c}}_{E_i}$, where **A** is the adjacency matrix for the network *G*. According to Bonacich [19], an optimal choice of *α*^−1^ is the largest eigenvalue of **A.**

- Brokerage: degree to which an actor occupies a brokerage position across all pairs of alters.

We also computed edge betweenness centrality which extends from the notion of vertex centrality by assigning to each edge a value reflecting the number of shortest paths traversing that edge. We calculated edge betweenness to assess which co-authorship collaborations are important for the flow of information. In the result section, we present the 10 most important collaborations in the malaria co-authorship network.

### Characterizing network cohesion

The extent to which subsets of authors are cohesive with respect to their relation in the co-authorship network was assessed through network cohesion. Specifically, we determined if collaborators (co-authors) of a given author tend to collaborate as well, and what subset of collaborating authors tend to be more productive in the network. While there are many techniques to determine network cohesion, we chose local triads and global giant components. In addition, we conducted cliques detection and clustering or communities detection on the network:

- Cliques: According to Kolaczyk and Csárdi [21], cliques are defined as complete subgraphs such that all vertices within the subset are connected by edges. We computed the number of maximal cliques and assessed their size.
- Density: Defined as the frequency of realized edges relative to potential edges, the density of a subgraph *H* in *G* provides a measure of how close *H* is to be a clique in *G*. Density values vary between 0 and 1:

4 $$ den(H)=\frac{\mid {E}_H\mid }{\mid {V}_H\mid \left({V}_H-1\right)/2} $$

- Relative frequency: we assess the relative frequency of *G* by computing its transitivity defined as:

5 $$ c{l}_T=\frac{3{\tau}_{\Delta}(G)}{\tau_3(G)} $$

where *τ*_Δ_(*G*) is the number of triangles in *G*, and *τ*_3_(*G*) is the number of connected triples (sometimes referred to as 2-star).

This measure is also referred to as the fraction of transitive triples. It represents a measure of global clustering of *G* summarizing the relative frequency with which connected triples close to form triangles [21].

- Connectivity, Cuts, and Flows: We investigated the concepts of vertex and edge cuts derived from the concept of vertex (edge) connectivity. The vertex (edge) connectivity of a graph *G* is the largest integer such that *G* is k-vertex- (edge-) connected [21]. These measures helped assess the information flow in the network. Since co-authorship networks are undirected graphs, the concept of weak and strong connectivity was irrelevant in this study. A graph *G* is said to be connected if every vertex in *G* is reachable from every other vertex. Usually, one of the connected components dominate the others, hence the concept of giant component.
- Graph Partitioning: Regularly framed as community detection problem, we applied graph partitioning to find subsets of vertices that demonstrate a ‘cohesiveness’ with respect to their underlying relational patterns. Cohesive subsets of vertices generally are well connected among themselves and are well separated from the other vertices in the graph. Two established methods of graph partitioning are Hierarchical clustering (agglomerative vs divisive) and Spectral clustering [21]. In this study, we applied agglomerative Hierarchical Clustering to the co-authorship network.

### Mathematical modeling

The purposes of network graph modeling are to test significance of the characteristics of observed network graphs, and to study proposed mechanisms of real-world networks such as degree distributions and small-world effects [21]. A model for a network graph is a collection of possible graphs $\mathcal{G}$ with a probability distribution ℙ_*θ*_ defined as:

6 $$ \left\{{\mathbb{P}}_{\theta }(G),G\ \epsilon\ \mathcal{G}:\theta\ \epsilon\ \varTheta \right\} $$

where *θ* is a vector of parameters ranging over values in *Θ*.Given our observed malaria co-authorship network graph *G*^*obs*^ and some structural characteristics *η*(·), our goal is to assess if *η*(*G*^*obs*^) is unusual. We then compare *η*(*G*^*obs*^) to collection of values $\left\{\eta (G):G\in \mathcal{G}\right\}$. If *η*(*G*^*obs*^) is too extreme with respect to this collection, then we have enough evidence to assert that *η*(*G*^*obs*^) is not a uniform draw from $\mathcal{G}$.Given the computationally expensive calculations involved in modeling in general, and the expected large size of our network, we parallelized all the processings.We applied different mathematical models for network graphs including:

- Classical Random Graph Models: First established by Erdős and Rényi [22–24], it specifies a collection of graphs $\mathcal{G}$ with a uniform probability ℙ(·) over $\mathcal{G}$. A variant of this model called the Bernoulli Random Graph Model was also defined by Gilbert [25].
- Generalized Random Graph Models: These models emanated from the generalization of Erdős and Rényi’s formulation, defining a collection of graphs $\mathcal{G}$ with prespecified degree sequence.
- Mechanistic Network Graph Models: These models mimic real-world phenomena and include Small-World Models commonly referred to as “six-degree separation”. It was introduced by Watts and Strogatz [26] and have since received a lot of interests in the existing literature especially in Neuroscience. Small-world networks usually exhibit high levels of clustering and small distances between vertices. Examples of known small-world networks include the network of connected proteins or the transcriptional networks of genes [27]. A variant of Small-World models is the Preferential Attachment Models defined based on the popular principle of “the rich get richer”. Examples of Preferential Attachment networks include that of World Wide Web [28] and the scientific citation network [29, 30]. An important characteristic of these models is that as time tend to infinity, there degree distribution tends to follow a power law.

For each mathematical model, we ran 1000 Monte-Carlo based simulations. We then compared the observed characteristics to the simulated ones thanks to a sample Student’s t-test. Characteristics we assess significance for are the average shortest paths, the clustering coefficient and the number of communities detected by the hierarchical clustering methods.

## Results

### Data collection

Of all the different queries formulated, the WOS query “TOPIC: (malaria) OR TOPIC: (mosquito) OR TOPIC: (anopheles) OR TOPIC: (plasmodium) OR TOPIC: (net) OR TOPIC: (vector) Refined by: COUNTRIES/TERRITORIES: (BENIN)” returned 630 records. After a rigorous screening process carried out by all the authors, 424 documents met the selection criteria. On average, there was 10.67 authors per published document.

After the Author Name Disambiguation, we identified 1792 unique authors with a precision of 99.87% and a recall of 95.46%. The generated multigraph co-authorship network therefore contained 1792 vertices (authors) and 116,388 parallel edges (collaborations). Each vertex (author) in the network has 2 attributes: name and a unique identification number. Each edge has 8 attributes: key, subject, abstract, year, wosid (Web of science Identification number), journal, title and doi (digital identifier object).

### Descriptive data analysis

**Fig. 1:**
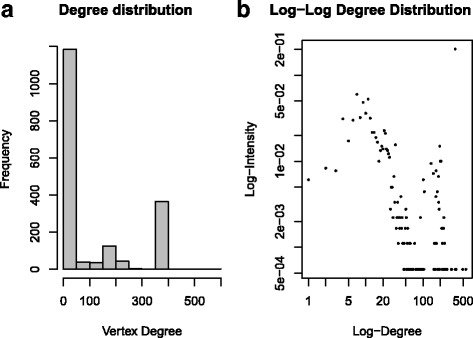
Degree distribution of the Malaria Co-authorship network

**Fig. 2:**
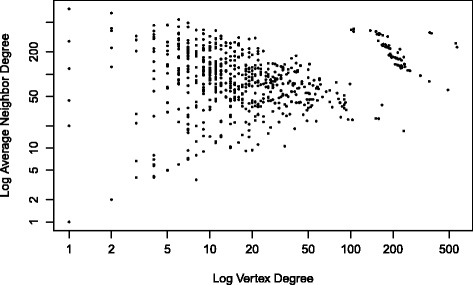
Log-Average Neighbor degree Distribution

The degrees of the multigraph network range between 1 and 1338 with an average degree distribution of 106.46. We noted in addition, a substantial number of vertices with low degrees (Fig. 1). There was also a non-trivial number of vertices with higher order of degree magnitudes. A log scale distribution of the degrees demonstrate that the vertex degrees tend to follow a heavy-tail distribution (Fig. 2).

After we convert the multigraph network in a weighted graph, it results in a simple graph of 1792 vertices and 95,787 weighted edges. Mean Closeness centrality ranges between 3.118 × 10^−7^ and 5.152 × 10^−6^ with a median of 5.112 × 10^−6^. This measure suggests a highly right-skewed distribution. Betweenness measures range between 0 and 245,600 with a median of 1985. A network visualization with the vertices’ size proportional to betweenness centrality measures clearly reveals the presence of broker authors (Table 1). The median Eigenvectors median is 0.005 and a mean of 0.09. Eigenvectors measures reveal the presence of multiple cluttered authors suggesting the presence of closed collaboration groups. Table 1 presents a list of the 10 authors with the highest Eigenvectors values.

**Table 1:** List of the most important authors and collaborations in the Malaria Co-authorship Network

Top 10 Brokers
MASSOUGBODJI ACHILLE
HAY SIMON I
KAREMA CORINE
SANNI AMBALIOU
KENGNE ANDRE PASCAL
AKOGBETO MARTIN
NDAM NICAISE TUIKUE
MALIK ELFATIH M
DABIRE K ROCH
DELORON PHILIPPE
Top 10 most connected authors (Top 10 network hubs)
MASSOUGBODJI ACHILLE
KAREMA CORINE
GONZALEZ RAQUEL
MENENDEZ CLARA
DALESSANDRO UMBERTO
OGUTU BERNHARDS R
FAUCHER JEANFRANCOIS
BASSAT QUIQUE
MARTENSSON ANDREAS
HAY SIMON I
Top 10 most important edges for information flow
DABIRE K ROCH _ KENGNE ANDRE PASCAL
BALDET THIERRY _ KENGNE ANDRE PASCAL
AKOGBETO MARTIN _ MALIK ELFATIH M
AVLESSI FELICIEN _ MOUDACHIROU MANSOUROU
AKOGBETO MARTIN _ AVLESSI FELICIEN
MASSOUGBODJI ACHILLE _ RAHIMY MOHAMED CHERIF
DIABATE ABDOULAYE _ KENGNE ANDRE PASCAL
GARCIA ANDRE _ SANNI AMBALIOU
KAREMA CORINE _ MALIK ELFATIH M
HAY SIMON I _ MALIK ELFATIH M
Weak articulation points
NOEL VALERIE
DJOGBENOU LUC
ZOHOUN I
SANNI AMBALIOU
EDORH ALEODJRODO PATRICK
ALLABI AUREL
HOUNKONNOU MAHOUTON NORBERT
FAYOMI BENJAMIN
KINDEGAZARD DOROTHEE A
DJOUAKA ROUSSEAU
RAHIMY MOHAMED CHERIF
BALDET THIERRY
DOSSOUGBETE L
GARCIA ANDRE
MASSOUGBODJI ACHILLE
AKOGBETO MARTIN

The computation of edge betweenness identifies co-authorship collaborations that are important for the flow of information. In Table 1, We present the top 10 most important collaborations for the flow of information in the Malaria Co-authorship network in Benin.

### Network cohesion

A total of 365 maximal cliques are identified in the network among which 9 cliques of size 2, 14 cliques of size 3, 155 cliques of size 8, and 142 cliques of size 7. Larger maximal cliques sizes range from 102 authors to 365 authors and are all found once across the network.

The malaria co-authorship network has a density of 0.0596 and a transitivity of 0.965 indicating that 96.5% of the connected triples in the network are close to form triangles. The transitivity metrics is a measure of the global clustering of the network.

**Fig. 3:**
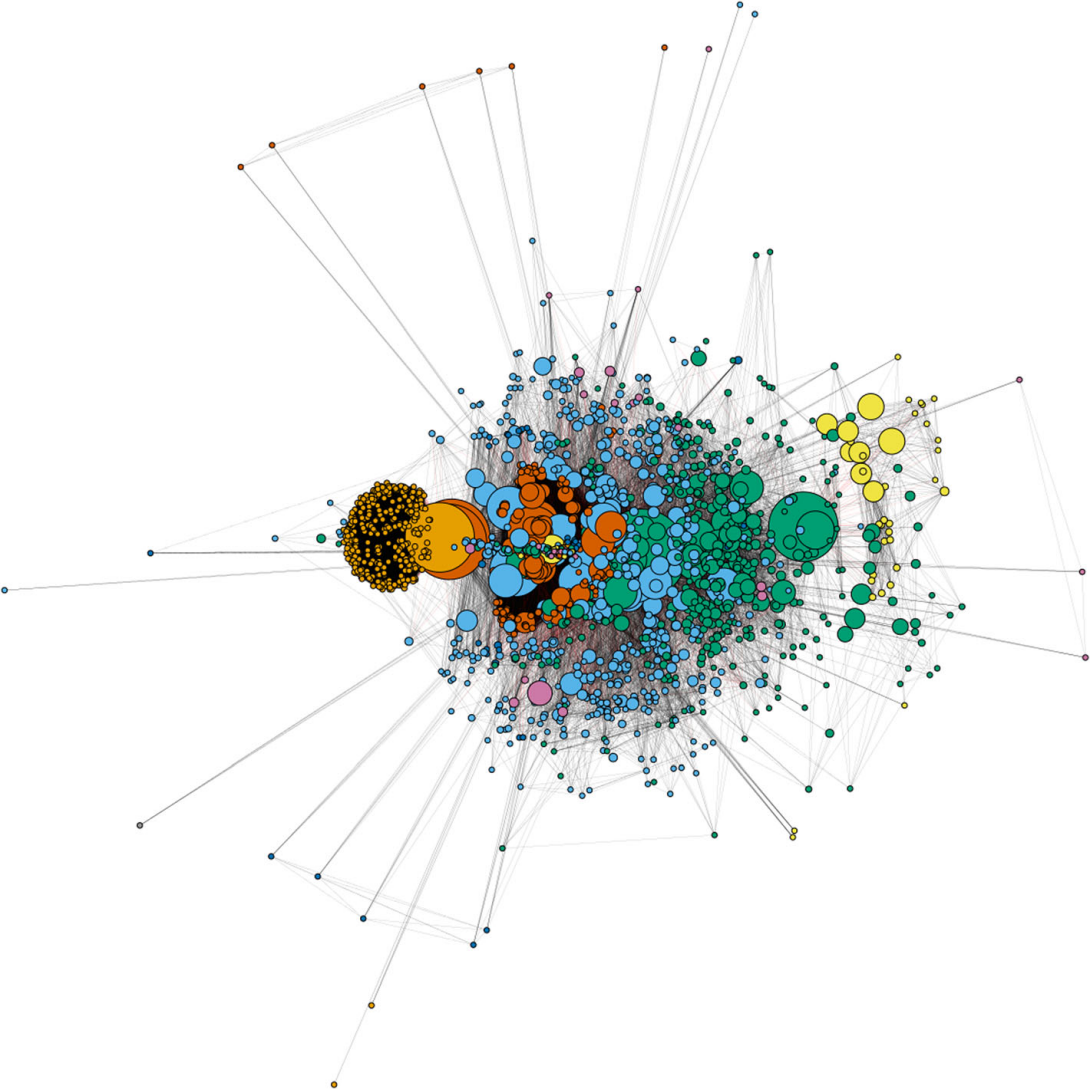
Malaria Co-authorship network – Main component. Authors (vertices) of the same color belong to the same research community or cluster

The network is not connected and a census of all the connected components within the network reveals the existence of a giant component that dominates all the other connected components. This giant component includes 94% (1686 vertices) of all the vertices in the network with none of the other components alone carrying less than 1% of the vertices in the network (Fig. 3).

The assessment of information flow in the network via cut vertices reveal the existence of 16 authors as the most vulnerable vertices in the network. Table 1 lists the authors that constitute the weak articulation points in the malaria co-authorship network. Cut vertices are crucial to the sustainability of networks [21].

The agglomerative hierarchical clustering method identifies 23 research communities (or clusters) in the network. Sizes of the clusters range between 2 and 570 with large research communities containing between 202 and 569 authors. Medium size research communities contain between 10 and 62 authors. Only seven out of the 23 research communities identified are part of the giant component. Figure 3 displays the giant component of the network with each different colors representing each of the seven research communities.

### Mathematical modeling

**Fig. 4:**
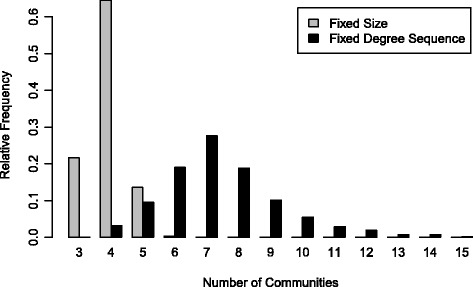
Monte-Carlo simulations: Number of detected communities by the random graph models

The hierarchical clustering method of community detection algorithm has identified 23 different clusters/communities in the co-authorship network out of which seven form a giant component. One of the question of interest in this section is whether the number of communities detected is expected or not. We performed 1000 Monte Carlo based simulations to test the significance of this observed characteristics on the malaria co-authorship network. Figure 4 clearly demonstrates that the number of communities detected is unusual from the perspective of both Classical random graphs and generalized random graphs (*p*-value < 0.0001). From the Classical random graph model, the expected number of communities is 3.934 (95%CI: 3.90–3.97). Similarly, the expected number of communities from the generalized random graph model is 7.501 (95%CI: 7.39–7.61).

**Fig. 5:**
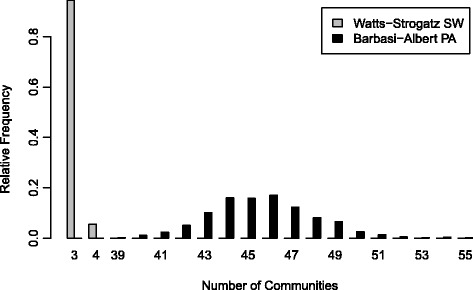
Monte-Carlo simulations: Number of detected communities by the Watts-Strogatz and the Barabási-Albert models

Figure 5 displays the number of detected research communities using the Barabási-Albert’s preferential attachment and the Watts-Strogatz models. Supprisingly enough, the observed number of communities is also extreme per both models (*p*-value < 0.0001). The expected number from the Watts-Strogatz model simulations is 3.056 (95%CI: 3.04–3.07) and 45.569 (95%CI: 45.42–45.72) from the Barabási-Albert model simulations.

We also compared the clustering coefficient and the average shortest-path length. The observed clustering coefficient is 0.9645. Surprisingly, there is substantially more clustering in our malaria co-authorship network than expected from all 4 mathematical models (*p*-value < 0.0001). The expected clustering coefficient is 0.0596 (95%CI: 0.05963068–0.05964648) and 0.4334 (95%CI: 0.4333912–0.4334522) respectively for the classic random graph and the generalized random graph models. Similarly, The Watts-Strogatz Small World model expected clustering is 0.7464 (95%CI: 0.7464326–0.7464356).

We observed an average shortest-path length of 2.99 in the malaria co-authorship network. This observed shortest-path length is significantly larger than what is expected from the random graph models (p-value < 0.0001) and significantly lower than what is expected from Watts-Strogatz small world model and the Barabási-Albert preferential attachment model (p-value < 0.0001).

The average shortest-path length is 1.94 (95%CI: 1.941955–1.941960) and 2.26 (95%CI: 2.259468–2.259586) respectively for the classic random graph and the generalized random graph models. For the Watts-Strogatz small world and the BarabÃ¡si-Albert models, the average shortest-path length is respectively 3.83 (95%CI: 3.81–3.86) and 9.17 (95%CI: 9.14–9.21).

All simulations were also performed on the giant component of the network and led to similar outcomes.

## Discussion and conclusion

This study provides insights in the structural characteristics of the malaria co-authorship network in Benin over a relatively long period. The 20 years of data collected coincides with the onset of active malaria research from 1996 until December 2016 in the country. The significant increase in malaria research and collaborations (Table 2) between the authors over the years is an expected finding given the regain and renewed interest in malaria control and elimination goals set forth [8, 31]. This research shows that the mechanism underlying the formation of the malaria co-authorship network in Benin is not random. It further demonstrates that the malaria research collaboration network in Benin is a complex network that seems to display small-world properties (often referred to as “six degrees of separation”).

**Table 2:** Distribution of published documents, authors and collaborations from January 1996 to December 2016

Year	Publications	Vertices (Authors)	Edges (Collaborations)
1996	3	16	68
1997	2	14	46
1998	1	2	1
1999	4	14	33
2000	1	9	36
2002	2	11	25
2003	2	9	16
2004	10	44	159
2005	16	62	285
2006	3	11	21
2007	22	112	809
2008	19	103	668
2009	27	136	704
2010	49	223	1853
2011	45	236	2100
2012	44	249	1759
2013	49	249	1917
2014	57	731	69,634
2015	41	453	25,302
2016	27	328	10,952
Aggregate	424	1792	116,388

The non-trivial number of authors with higher order of magnitudes confirms the presence of closed research groups where collaborative research likely happens only among members. In other words, interdisciplinary collaboration tends to occur at higher levels between prolific researchers with the majority of the collaborations happening between researchers from the same scientific communities. Prominent authors with important collaborations tend to collaborate with similar authors, young or less prolific authors tend to collaborate with both prolific authors and authors with very few collaborations. Similar findings were reported by Janet Okamoto [32] who studied scientific collaboration on a much smaller scale. Key brokers facilitate scientific collaborations within and outside their scientific community [33]. Betweenness centrality measures identifies such brokers who are important hubs for inter and transdisciplinary research. Many of the main brokers proved to also be the most connected and the most central authors confirming the presence of long publishing tenure authors in our network [34]. The flow of information in the malaria co-authorship in Benin is slow as it only relies on 16 authors representing less than 1% of all the authors in the network. Such a low information flow was also reported by Salamatia and Soheili [35] in a 2016 study on a co-authorship analysis of Iranian researchers in the field of violence. Generally, the most important authors in a co-authorship network are the ones with the highest degree of collaborations [36, 37]. However, to the long-term substainability of the malaria research network in Benin, The 16 authors identified as cut vertices are the most important authors. In other words, the removal of less than 1% of the authors from the network would lead to its collapse. Such a collapse would undoubtedly be detrimental to the future of malaria research in Benin. This finding clearly confirms the conclusion of Toivanen and Ponomariov [38] that the African research collaboration network is vulnerable to structural weaknesses and uneven integration.

Small-world networks are known to have small shortest path distance and a high clustering coefficient. Although our network seems to display such properties, the Monte-Carlo simulations revealed that the observed network has unexpected properties compared to classic small-world networks. A study of co-authorship network conducted on Chagas disease has found similar findings [13]. Unlike our study, the authors of this study did not deepen their analysis to confirm the small-world nature of their observed network. Other mechanisms such as preferential attachement have been found to explain the structure of international scientific collaboration network [39]. Unlike those studies, our network displayed unexpected properties that are more extreme that the 4 mathematical models we simulated. Our network has significantly larger shortest path distance and significantly higher clustering than expected from the 4 mathematical models presented here. One observation we are sure of is that none of the random graph models used here tend to explain the growth and the structure of the malaria co-authorship network in Benin. We therefore claim without any doubt that the structure and growth of our network is not random confirming the presence of hidden factors explaining the current structure of the network. Assessing such factors and the extent to which they influence scientific collaborations is important for the future of malaria research and its long-term sustainability. Unfortunately, none of the proposed models seem to accurately describe the observed structure of the network. This is why we believe that Advanced analyses involving statistical modeling are needed to better explain the structure of this network. In addition, unlike mathematical modeling, statistical modeling allow model fitting to the observed network [21, 40].

Our research has strengths. Unlike most studies on co-authorship analysis, it applies not only descriptive methods but also robust network analysis methods such as inferential methods like Monte-Carlo simulations. We test significance of the properties of our network to accurately understand its structure. Our data mining strategy involved a robust machine learning algorithm that helped address the crucial issue of the disambiguation of authors names and assign a unique identification to each of them. This technique maintained a good quality of the data collected throughout the pre-processing and analysis steps. To the best of our knowledge, our study is the first to describe the malaria research collaborations network via co-authorship network analysis in Benin.

The fact that our study collected data only from the Web Of Science can be considered as an important limitation of this study. However, according to Falagas and colleagues [41], who compared PubMed, Scopus, Web Of Science and Google Scholar in their paper, the Web Of Science appears as a reasonable scientific database source for our analysis. In addition, it proved to cover a wide range of both old and recently published papers. Falagas and colleagues [41] found PubMed to be the optimal choice in terms of scientific database. For that reason, we did run the same bibliographic search in PubMed. Unfortunately, the Web Of Science returns more relevant data than PubMed. Another limitation worth noting is that this study only looks at a snapshot of the malaria research network on a static fashion. There is also a need to apply dynamic statistical models such as Temporal Exponential Random Graph [42] and Dynamic Stochastic Block [43] modeling to better understand the temporal dynamic of collaboration formation in this network. Yet another limitation is inherent to the nature of all co-authorship studies. Collaborators, in a co-authorship network, do not often come from the same scientific discipline, or do not play the same roles on a particular research project. The data we collected did not allow us to accurately assess or even infer the disciplines each author came from or their specific contribution in the published document.

As malaria continues to be highly prevalent in Benin, it is essential to consolidate the knowledge generated from the numerous studies on the disease and reinforce the different communities involved in the research effort. Our results suggest that there is an urgent need to foster the malaria research network in Benin by continuously supporting, stabilizing the identified key brokers and most productive authors, and promoting the junior scientists in the field. Taking such measures will ultimately insure the long-term sustainability of the malaria co-authorship and collaboration network in Benin.

## Abbreviations

AND: Author Name Disambiguation
CI: Confidence Interval
US: United States
WOS: Web Of Science
